# Polymorphic variability in the 3' untranslated region (UTR) of *IL12B *is associated with susceptibility to severe anaemia in Kenyan children with acute *Plasmodium falciparum *malaria

**DOI:** 10.1186/1471-2156-12-69

**Published:** 2011-08-06

**Authors:** John M Ong'echa, Evans O Raballah, Prakasha M Kempaiah, Samuel B Anyona, Tom Were, Gregory C Davenport, Stephen Konah, John M Vulule, Collins Ouma, James B Hittner, Douglas J Perkins

**Affiliations:** 1 University of New Mexico Laboratories of Parasitic and Viral Diseases, Centre for Global Health Research, Kenya Medical Research Institute, Kisumu, Kenya; 2 Center for Global Health, Department of Internal Medicine, University of New Mexico School of Medicine, Albuquerque, NM, USA; 3 Department of Biochemistry and Biotechnology, Kenyatta University, Nairobi, Kenya; 4 Centre for Global Health Research, Kenya Medical Research Institute, Kisumu, Kenya; 5 Department of Psychology, College of Charleston, Charleston, SC, USA

## Abstract

**Background:**

*Plasmodium falciparum *malaria remains a leading cause of morbidity and mortality among African children. Innate immunity provides the first line of defence against *P. falciparum *infections, particularly in young children that lack naturally-acquired malarial immunity, such as the population examined here. Consistent with the fact that elevated interleukin (IL)-12 is an important component of the innate immune response that provides protective immunity against malaria, we have previously shown that suppression of IL-12 in African children is associated with the development of severe malarial anaemia (SMA). Since the role of *IL12B *variants in conditioning susceptibility to SMA remains largely unexplored, the association between a single nucleotide polymorphism (1188A→C, rs3212227), SMA (Hb<6.0g/dL), circulating IL-12p40/p70 levels, and longitudinal clinical outcomes in Kenyan children (n = 756) residing in a holoendemic falciparum malaria transmission area were investigated.

**Results:**

Multivariate logistic regression analysis in children with acute malaria (n = 544) demonstrated that carriers of the C allele had increased susceptibility to SMA (CC: OR, 1.674; 95% CI, 1.006-2.673; *P *= 0.047, and AC: OR, 1.410; 95% CI, 0.953-2.087; *P *= 0.086) relative to wild type (AA). Although children with SMA had lower IL-12p40/p70 levels than the non-SMA group (*P *= 0.037), levels did not differ significantly according to genotype. Longitudinal analyses in the entire cohort (n = 756) failed to show any significant relationships between rs3212227 genotypes and either susceptibility to SMA or all-cause mortality throughout the three year follow-up.

**Conclusion:**

The rs3212227 is a marker of susceptibility to SMA in children with acute disease, but does not appear to mediate functional changes in IL-12 production or longitudinal outcomes during the acquisition of naturally-acquired malarial immunity.

## Background

*Plasmodium falciparum *accounts for approximately 98% of the reported malaria cases in Africa and is a leading cause of morbidity and mortality among African children [1]. In areas with holoendemic *P. falciparum *transmission, such as western Kenya, severe malaria primarily manifests as severe malarial anaemia [SMA, haemoglobin, (Hb<6.0 g/dL)] with a peak incidence between 7-24 mos. of age [2]. Although SMA in western Kenya can occur in the presence or absence of high-density parasitaemia (HDP, ≥10,000 parasites/μL), the overall level of concomitant peripheral parasitaemia appears less important in determining anaemia severity than the presence of chronic or repeated infections that precipitously reduce Hb concentrations over time [3,4].

Interleukin (IL)-12 is a pro-inflammatory cytokine released by monocytes/macrophages, B cells, and dendritic cells as part of the host immune response to invading pathogens [5,6]. Studies in rhesus macaques demonstrated that subcutaneous injection of recombinant IL-12 prior to challenge with *P. cynomolgi *completely protected against infection, possibly through an interferon (IFN)-γ-dependent mechanism [7]. Our previous studies in Gabonese and Kenyan children with malaria showed that suppression of circulating IL-12 was associated with decreased Hb concentrations [8,9]. These results are consistent with studies in murine models showing that IL-12 protects against malaria by enhancing erythropoiesis and, thereby, reducing severe anaemia [10]. Thus, unlike a number of other cytokines that show divergent results in differing species, the association between reduced IL-12 production and enhanced malaria disease severity appears conserved across the phylogenetic spectrum.

IL-12 is a heterodimer composed of IL-12p35 and IL-12p40 subunits, encoded by *IL12A *and *IL12B *genes located on chromosomes 3p12-q13.2 and 5q31-33, respectively [11]. A number of single nucleotide polymorphisms (SNPs) have been identified in the *IL12B *gene, including an *IL12B *promoter polymorphism (IL12Bpro, rs17860508) and a *TaqI *polymorphism (an A to C transition at 1188, rs3212227) in the *IL12B *3' untranslated region [12,13]. Previous case-control studies revealed that the IL12Bpro polymorphism was associated with enhanced mortality and reduced peripheral nitric oxide (NO) production in Tanzanian children with cerebral malaria, whereas variation at this position had no relationship with disease outcomes in Kenyan children with severe malaria [14]. Investigation of *IL12B *polymorphisms in a family-based association study showed that IL12Bpro was associated with an increased risk of cerebral malaria in Malian children, while rs3212227 polymorphic variability had no significant relationship with susceptibility to cerebral malaria [15]. However, the rs3212227 polymorphism has been associated with susceptibility to the development of autoimmune diseases [16], type 1 diabetes mellitus [17], multiple sclerosis [18,19] lepromatous leprosy [20] and Chagas' disease [21]. *In vitro *investigations also suggest that differing rs3212227 genotypes functionally influence IL-12 production [13,22].

More recently, our studies among an adult Thai population showed that carriage of the rs3212227 CC genotype was associated with severe malaria [23]. A recent community-based longitudinal investigation in a paediatric population in western Kenya, however, observed no association between the rs3212227 variants and SMA, but several significant relationships between SMA and copy number variation in *IL12A *and *IL12RB1 *[24]. To further explore the role of rs3212227 variants in conditioning susceptibility to SMA, cross-sectional and longitudinal studies were performed in a comprehensively phenotyped paediatric population (n = 756) in Siaya District, western Kenya. We observed that among children presenting with acute falciparum malaria, carriers of the C allele had increased susceptibility to SMA compared to those with the wild type allele (AA), although IL-12p70 levels did not differ according to genotypes. In addition, longitudinal analyses did not show any significant relationships between the rs3212227 genotypes and either repeated episodes of SMA or all-cause mortality during the three year follow-up period.

## Methods

### Study participants

Recruitment took place at Siaya District Hospital (SDH), a rural government health facility in a holoendemic *P. falciparum *transmission area of western Kenya [4]. All study participants were from the Luo ethnic group. Further details about the study site and clinical manifestations of paediatric malaria in this geographic region can be found in our previous report [25]. A total of 756 children were recruited and included children presenting at the paediatric ward for treatment of febrile illnesses and those visiting the Mother and Child Health Clinic for childhood vaccinations. Enrolment was confined to those children presenting for their first documented 'hospital contact'. Children who had previously been hospitalized or had reported antimalarial use within two weeks prior to presentation at hospital were excluded from the study. None of the participants in the study had cerebral malaria or non-*P. falciparum *species of malaria.

For the cross-sectional analyses, children with *P. falciparum *malaria upon enrolment (Day 0, *n *= 544, 3-36 mos.), were stratified into two groups: non-SMA [Hb≥6.0 g/dL and *P. falciparum*-smear positive (density>1.0), n = 304 and SMA [Hb<6.0 g/dL and *P. falciparum*-smear positive (density>1.0), n = 240]. SMA was defined as Hb<6.0 g/dL (with any density parasitaemia) based on a previous longitudinal study examining the distribution of >14,000 Hb measurements in an age- and geographically-matched population in western Kenya [4]. This definition of SMA is appropriately defined by Hb distributions according to age, gender, and geographical context. Non-SMA was defined as Hb≥6.0 g/dL (with any density parasitaemia). In addition, to place the current findings into a global context, SMA (in the multivariate models) was also defined according to the World Health Organization (WHO) definition of SMA: Hb<5.0 g/dL with any density parasitaemia [26], while non-SMA was defined as Hb≥5.0 g/dL.

Since our previous investigations in this region demonstrated that HIV-1 status [27] and bacterial co-infection [28] enhance the development of anaemia in children with malaria, all study participants were tested for these co-pathogens (testing methods listed below). Pre- and post-HIV test counselling was provided for the parents/guardians of all study participants. Children were treated according to Ministry of Health (MoH), Kenya guidelines. Informed consent in the language of choice (i.e., English, Kiswahili, or Dholuo) was obtained from the parents/guardians of all participants. The study was approved by the ethical and scientific review committees at the Kenya Medical Research Institute and the Institutional Review Board at the University of New Mexico.

### Longitudinal follow-up

Following enrolment, parents/guardians were asked to bring their child back every 3 mos. throughout the 3 yr. follow-up period. Since the exact location of each child's residence was determined upon enrolment with a GIS surveillance system, children who did not present for the quarterly follow-up visit were located by the study team within two days following the missed visit. In addition, since children in this region experience multiple episodes of malaria, and other paediatric infectious diseases, parents/guardians were asked to return to the hospital during their child's febrile episode(s). At each acute and quarterly visit, all laboratory tests required for proper clinical management of the patients were performed, including complete haematological indices, malaria parasitaemia measures, and evaluation of bacteremia (if clinically indicated). In addition, mortality data was collected throughout the 3 yr. follow-up. Although most children die at their residence, visits by our study team determined mortality data for those children that did not return for their follow-up visits. Mortality data, along with clinical and laboratory measures were used to evaluate the association between the genotypes and SMA, as well as mortality over a three year follow-up period.

### Clinical laboratory measures

Venous blood samples (<3.0 mL) were collected into EDTA-containing Vacutainer^® ^tubes, prior to administration of any treatment interventions. Asexual malaria parasites (trophozoites) were counted against 300 leukocytes in peripheral blood smears stained with 10% Giemsa. Parasite densities were estimated using the following formula: parasite density/μL = white blood cell (WBC) count/μL × trophozoites/300. Complete haematological parameters were determined with a Beckman Coulter^® ^AcT diff2™ (Beckman-Coulter Corporation). Sickle-cell trait (HbAS) was determined by Hb electrophoresis according to the manufacturer's instructions (Helena Laboratories). HIV-1 exposure and HIV-1 infection were determined by serological and PCR testing, respectively, according to our published methods [27]. Trimethoprim-sulfamethoxazole was administered to all children that were positive for one or both serological HIV-1 tests. At the time of sample collection, none of the HIV-1(+) study participants had been initiated on antiretroviral treatment. For bacteremia testing, ~1.0 mL of whole blood was collected aseptically into sterile paediatric Isolator^® ^microbial tubes (Wampole™ Laboratories) and presence of blood-borne bacterial pathogens was determined according to our previous methods [28].

### rs3212227 SNP genotyping

DNA was extracted from buccal swabs using the BuccalAmp™ DNA extraction kit (Epicentre Biotechnologies). Genomic DNA was amplified using the Genomiphi DNA amplification kit (GE Healthcare, Life Sciences, Amersham), and the rs3212227 was genotyped using a Taqman^® ^5' allelic discrimination Assay-By-Design method according to manufacturer's instructions (Assay ID: C_2084293_10; Applied Biosystems).

### Determination of IL-12 production

Plasma samples were obtained from venous blood and snap-frozen at -80°C until use. IL-12p40/p70 concentrations were determined using the Cytokine 25-plex Ab Bead Kit, Hu (BioSource™ International) according to the manufacturer's protocol. Plates were read on a Luminex 100™ system (Luminex Corporation) and analyzed using the Bio-Plex Manager Software (Bio-Rad Laboratories). The level of detection for IL-12p40/p70 was >4.0 pg/mL.

### Statistical analyses

Statistical analyses were performed using SPSS (Version 15.0). Chi-square (χ^2^) analyses were used to compare proportions. Circulating levels of IL-12 were log-transformed towards normality prior to analyses. ANOVA tests were used to examine differences in IL-12 levels across the three genotypic groups. Differences in IL-12 levels between the non-SMA and SMA groups were determined using independent groups t-test. Deviations from Hardy-Weinberg Equilibrium (HWE) were tested using a web-based tool http://www.tufts.edu/~mcourt01/Documents/Court%20lab%20-%20HW%20calculator.xls. Cross-sectional associations between the rs3212227 genotypes and SMA in parasitaemic children were determined by a multivariate logistic regression model, controlling for the confounding effects of age, gender, HIV-1 status (this included both HIV-1 exposed and positive children), bacteremia, and sickle-cell trait (HbAS). In addition, hierarchical logistic regression was used to investigate the association between genotype and longitudinal outcomes (i.e., SMA and mortality). For each analysis, covariates (i.e., age, gender, sickle cell trait, HIV-1 and bacteremia status) were entered into block 1 and the genotype contrast was entered into block 2. Statistical significance was set at *P *≤ 0.050 for all tests.

## Results

### Demographic, parasitological, and clinical characteristics of parasitaemic children upon enrolment

To determine the impact of polymorphic variation at the rs3212227 locus on susceptibility to SMA in children with acute infection, children with *P. falciparum *infections (upon enrolment) were stratified into two clinical categories: non-SMA (Hb≥6.0 g/dL, n = 304) and SMA (Hb<6.0 g/dL, n = 240). The demographic, clinical, and laboratory characteristics of the parasitaemic study participants are summarized in Table 1. Although gender was comparable in the two groups (*P *= 0.268), children presenting at hospital with SMA were younger (mos., *P *< 0.001) and had lower axillary temperatures (°C, *P *< 0.001). Consistent with the *a priori *grouping based on Hb (g/dL) concentrations, Hb levels differed between the groups (*P *< 0.001). In addition, children presenting with SMA had leucocytosis relative to the non-SMA group (*P *< 0.001). Peripheral parasite density (/μL) and the geometric mean parasitaemia were comparable between groups (*P *= 0.446 and *P *= 0.678, respectively). However, the non-SMA group had an elevated prevalence of high-density parasitaemia (HDP, ≥10,000 parasites/μL, *P *= 0.057). Similarly, the non-SMA group had a higher proportion of children with sickle-cell trait (HbAS) compared to the SMA group (16.1% vs. 2.5%; *P *= 0.054).

**Table 1:** Demographic, parasitological, and clinical characteristics of the study participants upon enrolment

Characteristic	Non-SMA	SMA	*P*
Participants, *n*	304	240	N/A
Gender, female, *n *(%)	140 (46.1)	122 (50.8)	0.268*^a^*
Age, months	11 (10)	8 (7)	**<0.001*^b^***
Axillary temperature, °C	37.5 (1.7)	37.4 (1.5)	**<0.001*^b^***
Haemoglobin, g/dL	7.8 (2.7)	4.9 (1.4)	**<0.001*^b^***
White blood cells, (×10^9^/L)	10.9 (5.4)	13.1 (7.9)	**<0.001*^b^***
Parasite density,/μL	19,932 (40,097)	17,262 (41,739)	0.446*^b^*
Geomean parasitaemia,/μL	14,486	12,598	0.678*^c^*
High-density parasitaemia, *n *(%)	201 (66.1)	142 (59.2)	0.057^a^
Sickle-cell trait, *n *(%)	49 (16.1)	6 (2.5)	0.054^a^

Data are median values (interquartile range; IQR) unless otherwise noted. Children with *P. falciparum *malaria (n = 544) were categorized according to SMA status based on age- and geographically-appropriate Hb concentrations [i.e., Hb<6.0 g/dL, with any density parasitaemia) [4]] and non-SMA (Hb≥6.0 g/dL, with any density parasitaemia). High density parasitaemia (≥10,000 parasites/μL). *^a^*Statistical significance determined by Chi-square analysis. *^b^*Statistical significance determined by Mann-Whitney U test. *^c^*Statistical significance determined by independent groups t-test.

### Distribution of IL12B 3' UTR genotypes in children with malaria

Genotypes were generated using a Taqman^® ^5' allelic discrimination assay. Prevalence of rs3212227 genotypes in the parasitaemic children were 39.4% AA, 41.7% AC and 18.9% CC with overall allele frequencies of A = 0.60 and C = 0.40, respectively. The combined distribution of genotypes in the non-SMA and SMA groups displayed significant departure from Hardy-Weinberg Equilibrium (HWE, χ^2 ^= 9.078, *P *= 0.003). The distribution of rs3212227 genotypes in the non-SMA group was 43.4% AA, 40.5% AC, and 16.1% CC with allele frequencies of A = 0.64 and C = 0.36, respectively (Table 2). The genotypic distribution in the non-SMA group had significant departure from HWE (χ^2 ^= 4.796, *P *= 0.030). Genotypic distributions in SMA group were 34.2% AA, 43.3% AC, and 22.5% CC with allele frequencies of A = 0.56 and C = 0.44, respectively (Table 2). Departure from HWE did not reach significance in children with SMA (χ^2 ^= 3.536, *P *= 0.060). The overall distribution of genotypes between the non-SMA and SMA groups was statistically significant (χ^2 ^test, *P *= 0.048, Table 2).

**Table 2:** Distribution of *IL12B *3' UTR genotypes in children with *P. falciparum *malaria

*IL12B *3' UTR Genotypes	Non-SMA	SMA	*P^a^*
No. of participants, *n*	304	240	
AA	132 (43.4)	82 (34.2)	
AC	123 (40.5)	104 (43.3)	**0.048**
CC	49 (16.1)	54 (22.5)	
	P(A) = 0.64	P(A) = 0.56	

Data are presented as proportion of individuals [*n *(%)] within non-SMA and SMA groups. Children with *P. falciparum *malaria (n = 544) were categorized according to SMA status based on age- and geographically-appropriate Hb concentrations [i.e., Hb<6.0 g/dL, with any density parasitaemia) [4]] and non-SMA (Hb≥6.0 g/dL, with any density parasitaemia). *^a^*Statistical significance determined by Chi-square analysis. The overall distribution of genotypes between the non-SMA and SMA groups was statistically significant (χ^2 ^test, *P *= 0.048).

### Cross-sectional association between rs3212227 genotypic variants and SMA

To investigate the association between rs3212227 and susceptibility to SMA in children with acute disease, multivariate logistic regression analyses were performed controlling for the confounding effects of age, gender, sickle-cell trait, HIV-1, and bacteremia status [27-30]. Although heterozygous individuals (AC) had a 27% and 41% increased risk of developing SMA relative to homozygous A individuals (wild type, reference) using Hb cut-off criteria of 5.0 and 6.0 g/dL, respectively, these results were not statistically significant (*P *= 0.314 and *P *= 0.086, respectively; Table 3). However, homozygosity at the C allele was associated with a 51% increased risk of developing SMA using the WHO definition of disease (Hb<5.0 g/dL, OR, 1.512; 95% CI, 0.870-2.629; *P *= 0.142) and a 67% increased risk of SMA (Hb<6.0 g/dL, OR, 1.674; 95% CI, 1.006-2.673; *P *= 0.047) using the modified definition of SMA (Table 3). Additional analyses revealed that carriage of the C allele (AC + CC) was associated with 35% increased risk of developing SMA at Hb<5.0 g/dL (*P *= 0.170) and a 48% increased risk of developing SMA at Hb<6.0 g/dL (*P *= 0.034, Table 3). Although the CC vs. AC + AA demonstrated a trend towards increased risk in those with CC genotype relative to AC + AA groups, these findings were not statistically significant (Table 3). Further analyses stratifying the genotypes into different alleles revealed the same results for the A vs. C alleles and supported the observation that the presence of a C allele increased the risk of developing SMA. Taken together, these results demonstrate that carriage of the C allele is an important risk factor for susceptibility to SMA in children with acute disease using either the appropriately modified definition of SMA or the more globally employed definition of SMA set by the WHO.

**Table 3:** Relationship between *IL12*B 3' UTR genotypes and susceptibility to SMA

*IL-12B *3' UTR Genotype	OR	95% CI	*P*	OR	95% CI	*P*
	**SMA (Hb<5.0 g/dL)**			**SMA (Hb<6.0 g/dL)**		

AA	reference			reference		
AC	1.268	0.799-2.011	0.314	1.410	0.953-2.098	0.086
CC	1.512	0.870-2.629	0.142	1.674	1.006-2.673	**0.047**
CC vs. AC + AA	1.335	0.816-2.185	0.249	1.370	0.880-2.133	0.826
AA vs. AC+CC	1.346	0.881-2.058	0.170	1.479	1.031-2.123	**0.034**

Parasitaemic children (*n *= 544) were categorized according to the WHO SMA definition [i.e., Hb<5.0 g/dL, with any density parasitaemia [26]] and the age- and geographically-appropriate definition of SMA [(Hb<6.0 g/dL, with any density parasitaemia [4]]. Odds Ratio (OR) and 95% confidence interval (CI) were determined using multivariate logistic regression controlling for age, gender, HIV-1, bacteremia, and sickle-cell trait (HbAS) status.

### Functional relationship between rs3212227 genotypes and IL-12 production

**Figure 1:**
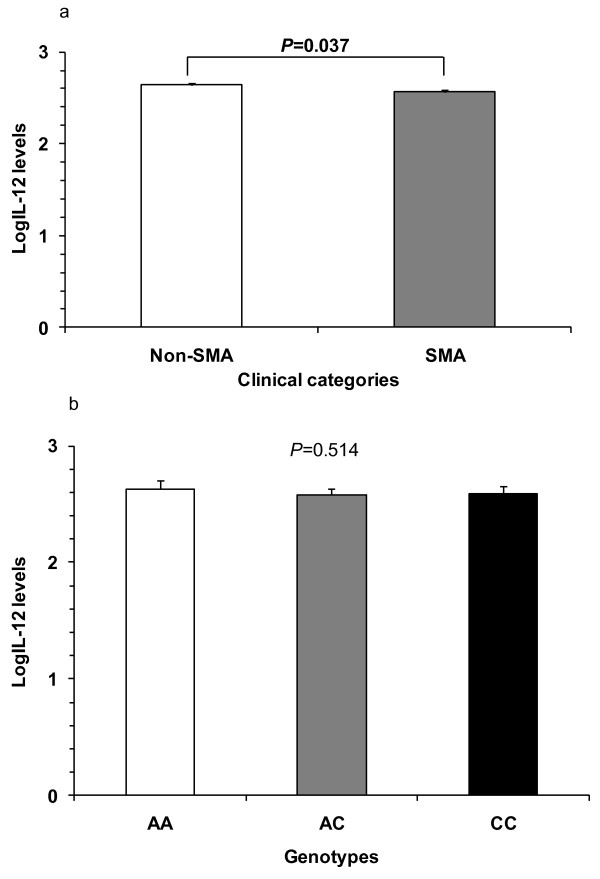
**IL-12p70 production based on anaemia severity and genotypes**. **(A) **The association between circulating IL-12p40/p70 levels and anaemia severity in parasitaemic children with SMA (n = 95) and non-SMA (n = 95) was determined. Bar-charts represent the means, and whiskers show the standard error of mean (SEM). Statistical significance was determined at *P *≤ 0.05 by independent groups t-test. Children with SMA had significantly lower circulating IL-12p40/p70 levels than the non-SMA group (*P *= 0.037). **(B) **Circulating IL-12p40/p70 levels in children (n = 190) with *P. falciparum *malaria were measured using the Human Cytokine 25-Plex Antibody Bead Kit and stratified according to genotype. Bar-charts represent the means, and the whiskers show the standard error of mean (SEM) for AA (n = 69) 2.625 ± 0.035, AC (n = 82) 2.576 ± 0.027, and CC (n = 39) 2.590 ± 0.030 genotypes. Differences were considered significant at *P *≤ 0.05 (ANOVA). IL-12p40/p70 levels were not significantly different across the genotypic groups (*P *= 0.514).

Prior to examining the functional association between circulating IL-12 levels and rs3212227 variants, IL-12 levels were log-transformed towards normality and compared between the non-SMA and SMA groups. Since bacteremia and HIV-1 infection could influence cytokine production in children with malaria, all co-infected children were excluded from the analysis. Children with SMA (n = 96) had significantly lower circulating IL-12p40/p70 levels [mean (SEM); 2.563 (0.023)] than the non-SMA (n = 98) group [2.638 (0.028), *P *= 0.037] (Figure 1a).

To determine if rs3212227 genotypes were associated with functional changes in IL-12, circulating concentrations of log-transformed IL-12p40/p70 levels were compared across the genotypic groups. Levels of IL-12p40/p70 were comparable in the groups (*P *= 0.514, ANOVA; Figure 1b), suggesting that variation at rs3212227 may be an important marker of disease severity, but may not be critical for regulating the overall production of IL-12 in children with acute malaria.

### Longitudinal relationship between the rs3212227, SMA, and mortality

After determining the relationship between the rs3212227 locus and susceptibility to SMA in children with acute malaria, hierarchical logistic regression was used to investigate the relationship between carriage of the different genotypes and longitudinal outcomes: SMA and mortality. Genotypic distributions in the entire cohort (n = 756) were: 40.3% (AA); 41.4% (AC); and 18.3% (CC). Hierarchical logistic regression modelling revealed that relative to the AA genotype (reference), neither the AC (β = -0.004, *P *= 0.963) nor CC (β = 0.005, *P *= 0.964) genotypes were associated with development of SMA over the three year follow-up period. The relationship between genotypes and mortality were also examined by hierarchical logistic regression. The mortality rate over the three years of follow up was 7.1% (54/756). Compared to the AA reference group, mortality over the follow-up period was not significantly different for the AC (β = -0.571, *P *= 0.130) and CC (β = -0.681, *P *= 0.181) genotypes. Further exploration by both Poisson and Negative Binomial regressions also failed to identify any significant relationships between the genotypes and either susceptibility to SMA or mortality (data not presented).

## Discussion

The pathophysiological basis of SMA is complex and poorly understood. One method for exploring the genes and gene pathways that condition susceptibility to SMA is to utilize a candidate gene approach for both cross-sectional and longitudinal investigations. This approach allows for an increased understanding of the mechanisms that confer protection against development of SMA once children become infected with falciparum malaria (acute disease), and also provides information about how polymorphic variants condition susceptibility to SMA and/or mortality throughout the critical phases of naturally-acquired immunity (longitudinal outcomes). We hypothesized that variation at rs3212227 locus may provide important information about the role of IL-12 in protecting against SMA.

Children with falciparum malaria underwent extensive clinical evaluations to rule out additional causes of anaemia so that they could be appropriately controlled for in the multivariate models. For example, since HIV-1 and bacteremia are some of the most common anaemia-promoting infectious diseases in African children [27-29], all study participants were tested for these co-pathogens. It is important to note that children exposed to HIV-1, yet lack definitively identifiable virus in their system, also have enhanced severity of anaemia when they acquire falciparum malaria [27]. Unlike previous studies, the multivariate models therefore controlled for HIV-1 exposure, HIV-1 positivity, and bacteremia. Since severe anaemia is the primary manifestation of severe malaria in holoendemic *P. falciparum *transmission regions, with cerebral malaria being a rare occurrence, this study specifically examined the role of rs3212227 in conditioning the development of severe anaemia and as such, it remains to be determined if results obtained here are applicable to areas with differing endemicities of malaria in which individuals develop severe disease manifestations apart from severe anaemia.

IL-12 plays a critical role in modulating the immune response by promoting CD4+ T cells and NK cells to differentiate towards a Th1 phenotype for production of IFN-γ, an important component of cell-mediated immunity that helps eliminate intracellular pathogens [31]. Accumulating evidence shows that IL-12 is a critical factor for augmenting protective immune responses against malaria in animal models [7,32,33]. Our previous studies showing that reduced IL-12p70 production is associated with enhanced severity of malaria in African children [8,34] illustrate the importance of IL-12 in human malaria as well. Consistent with these investigations, the current study demonstrated that children with acute malaria that develop SMA have significantly lower circulating IL-12p40/p70 levels than children that fail to progress to severe anaemia (i.e., the non-SMA group).

Previous examination of the rs3212227 polymorphism in Tanzanian children demonstrated that homozygous C individuals had higher mortality from cerebral malaria, whereas no significant associations were found between rs3212227 genotypes and severe malaria (defined as a mixed phenotypic outcome) in children from coastal Kenya [14]. Our recent study in Thai adults with falciparum malaria showed that although carriage of the rs3212227 CC genotype was enriched in the severe disease group, multivariate modelling did not reveal a significant association between the genotype and disease severity [23]. In addition, a comprehensive longitudinal study in an adjacent region to where the current study was conducted utilized fifty-five tagging single nucleotide polymorphisms (covering genes encoding *IL12A, IL12B*, *IL12RB1*, and *IL12RB2*) to demonstrate a number of important associations between genetic variation and SMA, of which rs3212227 did not emerge as significant [24]. However, results presented here clearly demonstrate that homozygosity at the C allele was associated with an increased risk of developing SMA in children with acute disease, while having no effect on longitudinal outcomes (i.e., SMA and mortality) over three years of follow-up. While the reason for differences in the predominately Luo ethnic populations examined in both studies is unclear, it may be attributable to the fact that we controlled for the influence of common paediatric infectious diseases, such as HIV-1 and bacteremia, that influence both anaemia and mortality throughout childhood. Furthermore, the distribution of genotypes in the combined clinical groups (non-SMA and SMA) displayed significant departure from HWE, possibly resulting from selection at the rs3212227 SNP due to 'enrichment' of malaria-infected individuals in the current study. Although not available for the current study, examination of the genotypes in a large number of non-malaria infected 'controls' would help to determine if the HWE deviation at rs3212227 observed in the current study is indeed due to malaria-mediated selection.

Since 3'UTRs contain regulatory elements that are highly conserved and control translational activity, variation within the 3'UTR can affect transcriptional stability and binding of translational proteins [35]. A previous investigation examining the rs3212227 in Turkish adults showed that individuals with the CC genotype produced the highest levels of IL-12p70 in cultured peripheral blood mononuclear cells (PBMC) [22,36], while another study in two ethnic groups from Bulgaria revealed that the AA genotype was associated with the highest PBMC IL-12p40 production [22,36]. However, recent studies in individuals with lepromatous leprosy and Chagas' disease failed to find any significant relationships between rs3212227 genotypes and IL-12p70 production [20,21]. Consistent with these investigations, stratification of children in the current study, according to genotypes, failed to show any significant differences in circulating IL-12p40/p70 levels across the groups. Moreover, examination of IL-12p40/70 in cultured PBMC from children with malaria in the current study also failed to show any significant association with rs3212227 genotypes (data not presented). The lack of a functional association between rs3212227 variants and IL-12 production reported here is supported by our *in silico *analysis (using the web site http://www.cbil.upenn.edu/cgi-bin/tess/tess) which revealed that the region encompassing the rs3212227 had no major transcription factor binding site changes that would be affected by SNP variation within this region. Although the exact reason for the lack of a functional association between rs3212227 genotypes and IL-12p40/p70 levels reported here remains to be determined, it is possible that additional polymorphic variants, such as IL12Bpro [14], or variation in other genes that influence IL-12 production (e.g. IL-10 and IL-4) may be responsible for mediating IL-12 generation [36,37].

Since the mechanism through which IL-12 provides protection against malaria in murine models appears to be related to the ability of IL-12 to promote IFN-γ and tumour necrosis factor (TNF)-α production that, in turn, provide enhanced NO generation [38], we examined the association between the rs3212227 variants and all three of these inflammatory mediators. However, circulating levels of IFN-γ, TNF-α, or NO were not significantly different upon stratifying into genotypic groups (data not presented).

Recent data from our laboratory, obtained in the same population of children, demonstrated that multi-site loci provide more informative information about malarial disease outcomes than information obtained for a single locus [39,40]. These studies further revealed that combinations of functional polymorphic alleles in haplotypes interact to modulate the individual effects of SNPs and variable number tandem repeats (VNTRs) [39,41]. As such, we are currently expanding the current study to include additional polymorphisms in the *IL12B *gene (and other immunoregulatory genes that influence IL-12), to delineate the effects of genetic variation in *cis*-regulatory genes, and the impact of haplotypes on the host immune response to malaria.

## Conclusions

Results presented here demonstrate that the rs3212227 is an important marker of susceptibility to SMA, but variation at this particular locus may not serve as a functional mediator of IL-12p40/p70 levels. Given that similar results were observed in individuals with lepromatous leprosy [20] and Chagas' disease [21], the CC genotype could be a marker of susceptibility to intracellular pathogens. Additional studies are required to confirm this hypothesis.

## Competing interests

The authors declare that they have no competing interests.

## Authors' contributions

JMO participated in the design and implementation of the genotyping assays, supervised the study and participated in writing the manuscript. EOR participated in genotyping experiments, data analyses and manuscript writing. PK participated in the genotyping assays and writing of the manuscript. SBA participated in the analyses and manuscript writing. TW participated in the analyses and manuscript writing. GCD participated in the analyses and manuscript writing. SK participated in the analyses and manuscript writing. JMV participated in the design, supervision of the study, and manuscript writing. CO participated in the analyses and manuscript writing. JBH participated in the design, analyses and manuscript writing. DJP conceived the study, participated in the design, implementation, analyses and manuscript writing. All authors read and approved the final manuscript.

## Authors' information

University of New Mexico Laboratories of Parasitic and Viral Diseases, Centre for Global Health Research, Kenya Medical Research Institute, Kisumu, Kenya (JMO, EOR, SBA, TW, SK, CO, and DJP); Center for Global Health, Department of Internal Medicine, University of New Mexico School of Medicine, Albuquerque, NM, USA (PK, JMO, GCD and DJP); Department of Biochemistry and Biotechnology, Kenyatta University, Nairobi, Kenya (EOR); Centre for Global Health Research, Kenya Medical Research Institute, Kisumu, Kenya (JMV); Department of Psychology, College of Charleston, Charleston, SC, USA (JBH).
